# Spontaneous Ovarian Hyperstimulation Syndrome in a Naturally Conceived Singleton Pregnancy With Familial Predisposition: A Case Report

**DOI:** 10.7759/cureus.115876

**Published:** 2026-09-06

**Authors:** Madinah Azeez, Victoria Porter

**Affiliations:** 1 Obstetrics and Gynecology, Bolton NHS Foundation Trust, Bolton, GBR

**Keywords:** ascites, ca125, case report, human chorionic gonadotropin, ovarian enlargement, ovarian hyperstimulation syndrome (ohss)

## Abstract

Spontaneous ovarian hyperstimulation syndrome (sOHSS) is an uncommon condition that can occur in naturally conceived pregnancies and may present a significant diagnostic challenge because its clinical and radiological features can resemble ovarian malignancy. We describe the case of a 30-year-old primigravida who presented in early pregnancy with abdominal pain and was found to have bilateral multicystic ovarian enlargement, ascites, and a markedly elevated cancer antigen-125 (CA-125) despite a viable singleton pregnancy conceived without fertility treatment. A detailed history revealed a strong familial pattern of sOHSS, raising suspicion of an inherited predisposition. Following multidisciplinary assessment, she was managed conservatively with close clinical, biochemical, and ultrasound surveillance, resulting in progressive resolution of ovarian enlargement and ascites with an ongoing uncomplicated pregnancy. This case highlights the importance of recognizing sOHSS as a differential diagnosis in natural conception pregnancies, obtaining a thorough family history, and adopting an appropriate multidisciplinary approach to avoid unnecessary intervention while achieving favorable maternal and fetal outcomes.

## Introduction

Ovarian hyperstimulation syndrome (OHSS) is a recognized complication of assisted reproductive technology (ART) resulting from an exaggerated ovarian response to gonadotrophin stimulation. It is characterized by bilateral cystic ovarian enlargement, increased vascular permeability, and third-space fluid accumulation, with clinical manifestations ranging from mild abdominal discomfort to severe disease complicated by ascites, thromboembolism, renal impairment, and, rarely, maternal mortality. Vascular endothelial growth factor (VEGF), released in response to human chorionic gonadotropin (hCG), is considered the principal mediator of the increased capillary permeability that underlies the syndrome [1,2]. The severity of OHSS is commonly described using the Golan classification, which categorizes the syndrome as mild, moderate, or severe according to ovarian enlargement, the presence of ascites, and associated systemic manifestations [3].

In contrast, spontaneous ovarian hyperstimulation syndrome (sOHSS) is exceptionally rare, occurring in the absence of ovulation induction or fertility treatment. Most reported cases occur during the late first trimester and have been associated with multiple gestation, gestational trophoblastic disease, severe hypothyroidism, and activating mutations of the follicle-stimulating hormone receptor (FSHR), which render the receptor abnormally sensitive to endogenous hCG [4-6]. Familial sOHSS is particularly uncommon and has been linked to heterozygous activating mutations of the FSHR gene, resulting in recurrent disease in naturally conceived pregnancies [5,6].

During early pregnancy, physiological ovarian enlargement may occur as a result of corpus luteum activity. However, marked bilateral multicystic ovarian enlargement accompanied by ascites is not a typical physiological finding and should prompt consideration of pathological causes, including OHSS [2,4].

The diagnosis of sOHSS may be challenging because its clinical and radiological features can closely mimic ovarian malignancy. Marked bilateral multicystic ovarian enlargement, ascites, and elevated serum cancer antigen-125 (CA-125) concentrations may initially prompt investigation for an ovarian neoplasm, particularly in women without a history of fertility treatment. Careful clinical assessment, serial imaging, biochemical investigations, and multidisciplinary team (MDT) review are therefore essential to establish the correct diagnosis and avoid unnecessary surgical intervention during pregnancy [2,7,8]. We present a rare case of sOHSS in a spontaneously conceived singleton pregnancy with a strong family history suggestive of a possible inherited predisposition.

## Case presentation

A 30-year-old primigravida woman presented to the Early Pregnancy Assessment Unit (EPAU) at approximately 11+1 weeks of gestation based on her last menstrual period (LMP) with generalized abdominal pain and light vaginal bleeding. Clinical examination demonstrated a closed cervix with no evidence of miscarriage. There was low clinical suspicion for ectopic pregnancy, and she was scheduled for ultrasound assessment.

The patient reattended three days later for a transvaginal ultrasound, which demonstrated a live, viable intrauterine pregnancy measuring 8+5 weeks of gestation (Figure 1). The gestational age was therefore revised to 8+5 weeks based on the ultrasound assessment. By the time of this assessment, the vaginal spotting had completely resolved, and the patient was asymptomatic, with no nausea or vomiting. Bilateral enlarged multicystic ovaries were identified, with a right ovarian volume of 301.6 cc (Figure 2) and a left ovarian volume of 217.2 cc (Figure 3). Ovarian tumor markers were discussed; however, the patient initially declined. 

**Figure 1:**
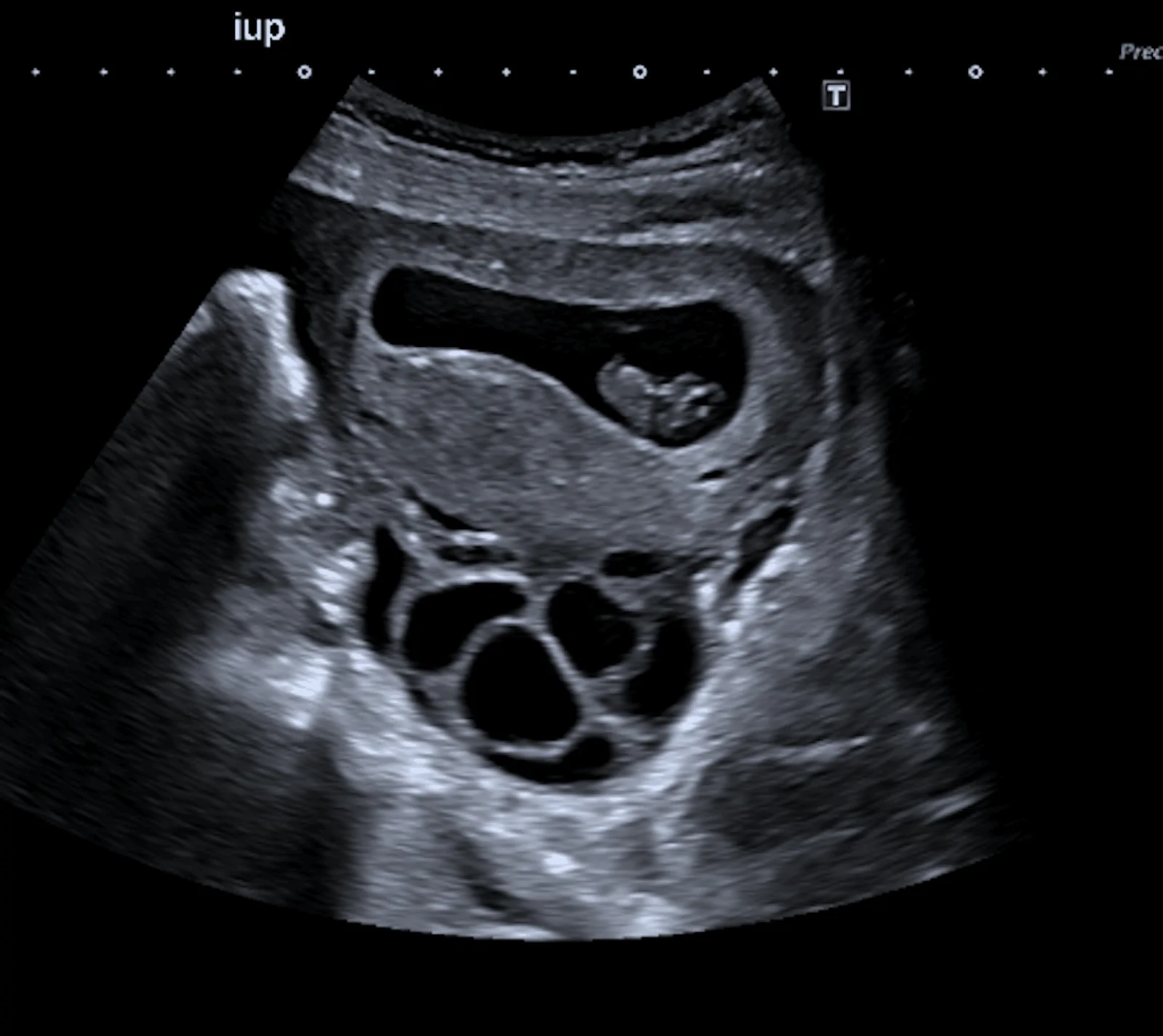
Initial ultrasound scan at 8+5 weeks confirming viable intrauterine pregnancy Viable single intrauterine fetus, measuring eight weeks and five days (CRL=20.5 mm), with a multicystic ovary. CRL: crown-rump length

**Figure 2:**
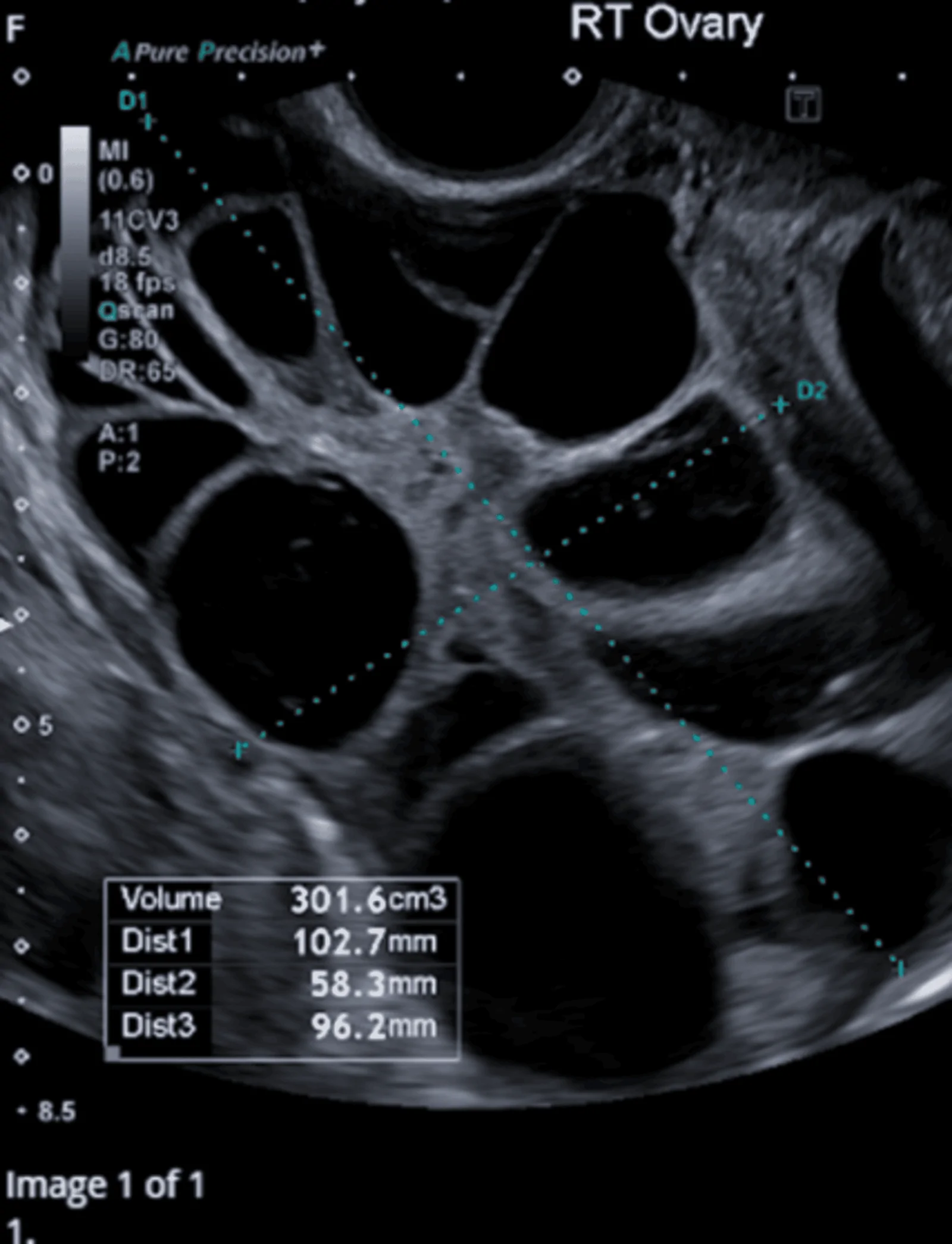
Right ovary at initial ultrasound scan (8+5 weeks) Right large ovary containing multiple cysts surrounded by fluid, with an ovarian volume of 301.6 cc. The appearance is suggestive of ovarian hyperstimulation syndrome.

**Figure 3:**
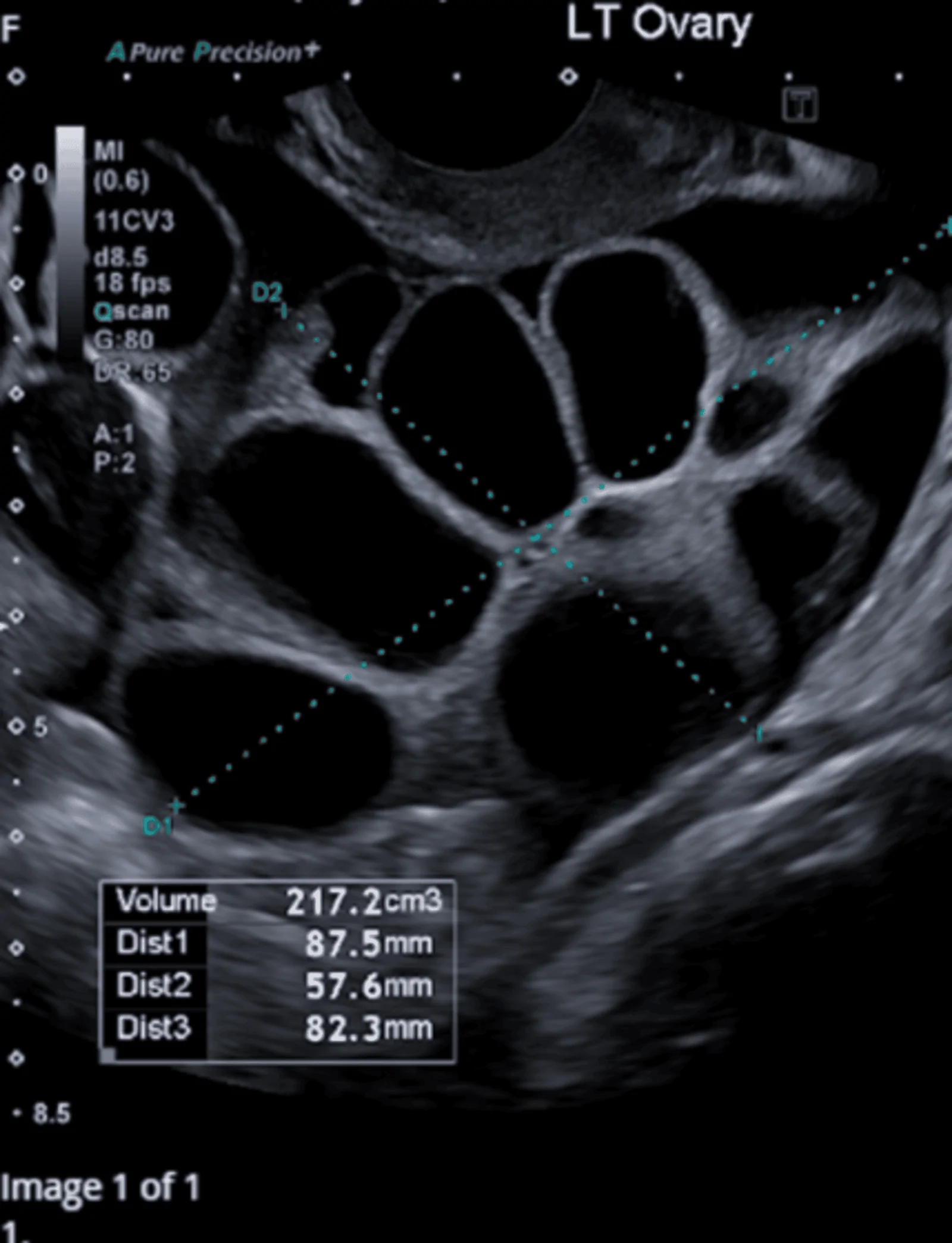
Left ovary at initial ultrasound scan (8+5 weeks) Left ovary with an ovarian volume of 217.2 cc demonstrating a multicystic ovary.

Further history revealed a strong family history of sOHSS occurring during naturally conceived pregnancies. Her sister had experienced sOHSS during each of her three naturally conceived pregnancies, with one episode requiring hospitalization. The patient had no history of assisted reproductive treatment or ovulation induction. This family history raised the possibility of an inherited predisposition to sOHSS, particularly involving activating mutations of the FSHR, although genetic testing was not performed during pregnancy.

Fifteen days after the initial ultrasound, at 11+0 weeks of gestation, repeat ultrasound demonstrated significantly enlarged bilateral multicystic ovaries, with ovarian volumes increasing to 824 cc on the right (Figure 4) and 429 cc on the left (Figure 5), accompanied by abdominal ascites. Figure 6 demonstrates an ongoing viable intrauterine pregnancy at 11+0 weeks. Despite these findings, the patient remained clinically well with minimal symptoms. 

**Figure 4:**
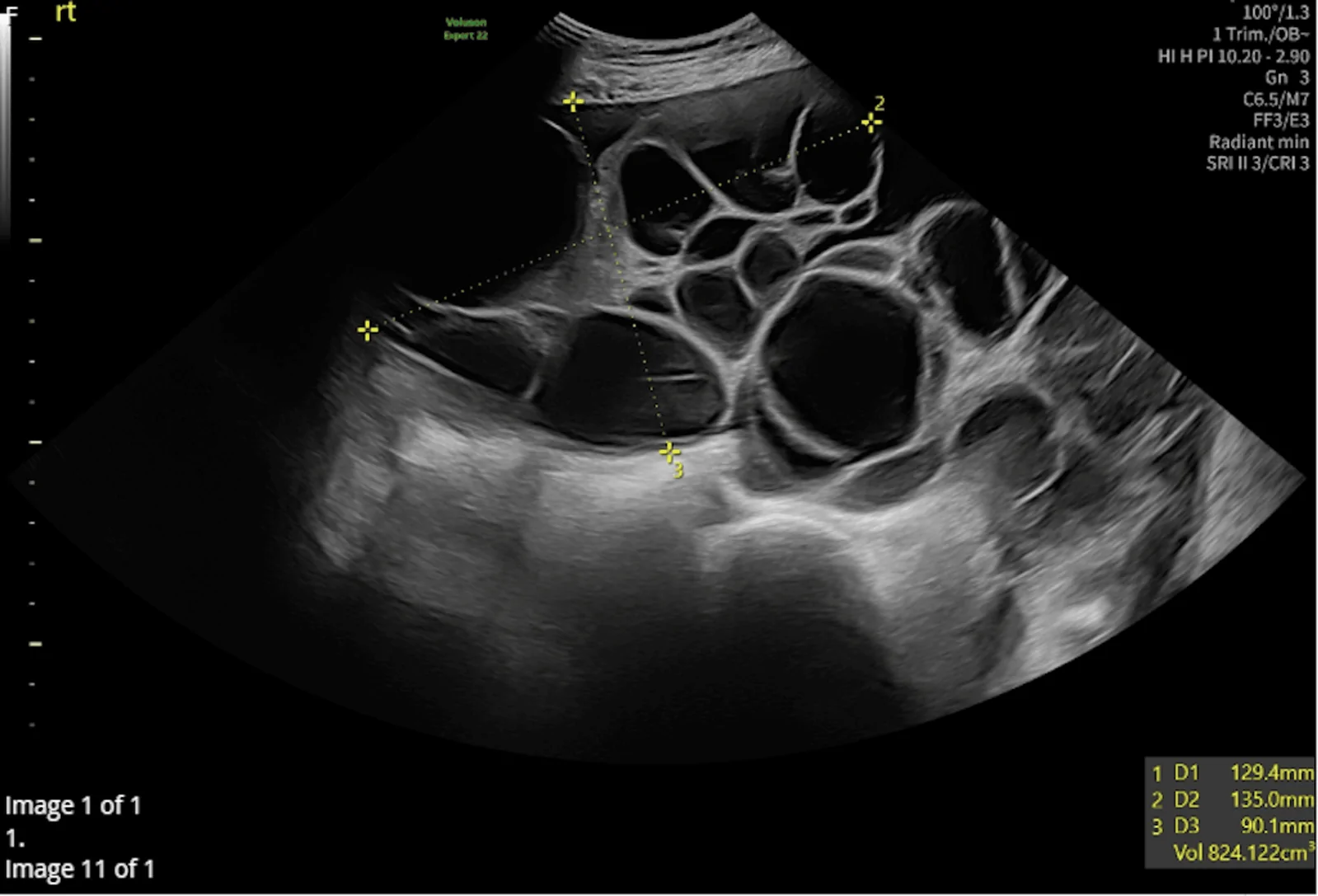
Right ovary on repeat ultrasound scan at 11+0 weeks Right ovary with ovarian volume of 824.122 cc demonstrating marked enlargement and ascites.

**Figure 5:**
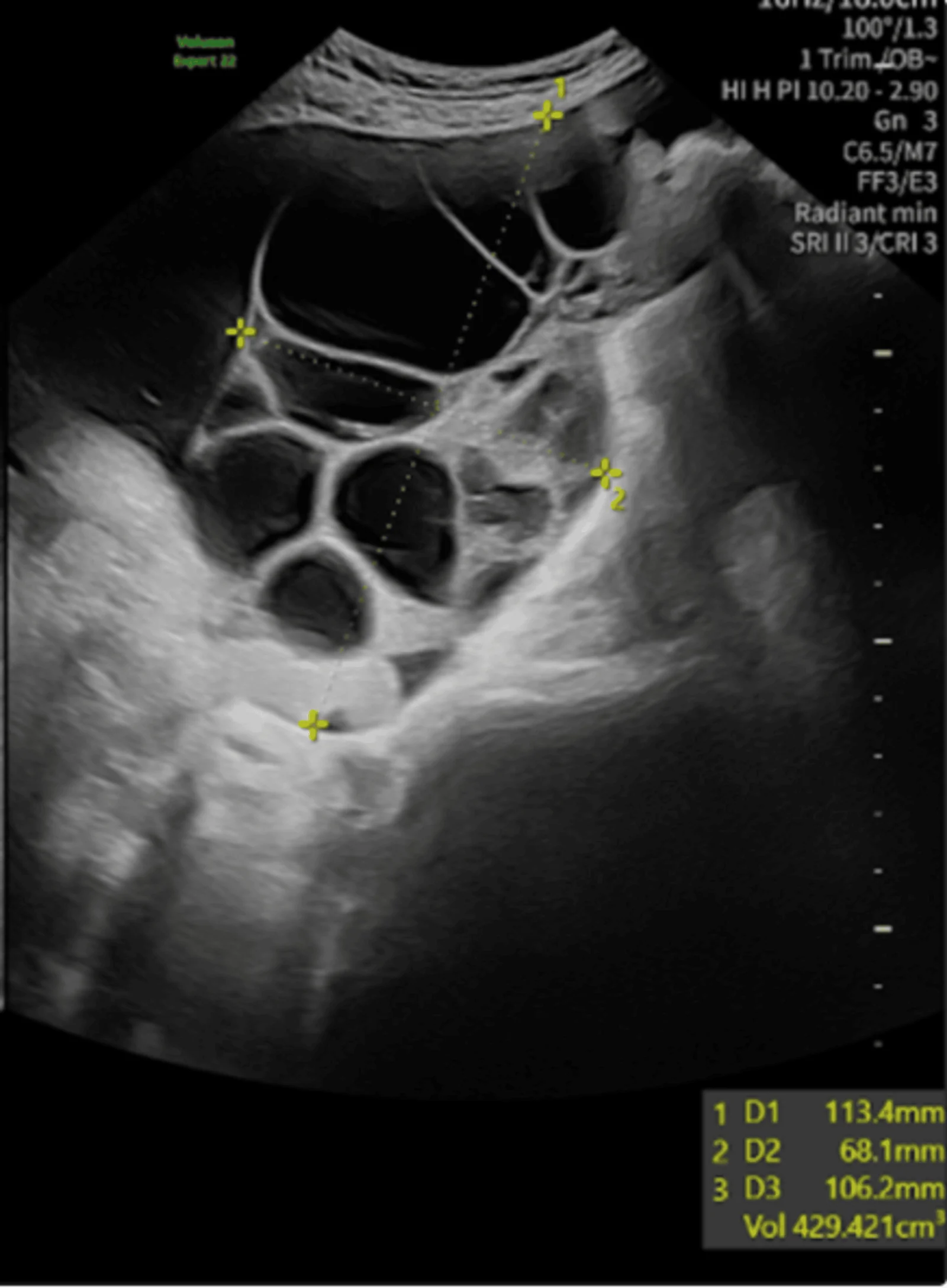
Left ovary on repeat ultrasound scan at 11+0 weeks Left ovary with ovarian volume of 429.421 cc demonstrating marked enlargement and ascites.

**Figure 6:**
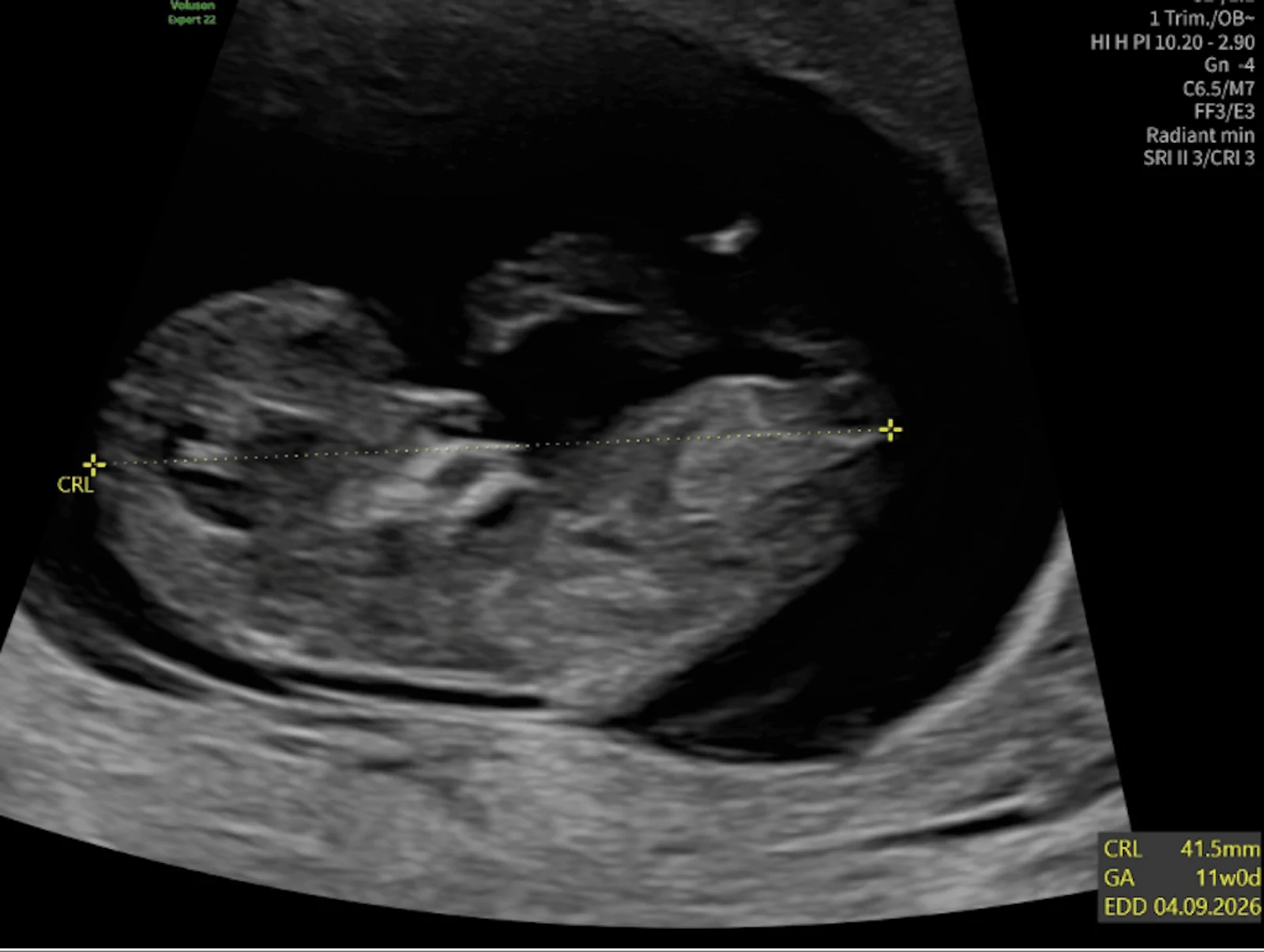
Ultrasound scan at 11+0 weeks confirming viable intrauterine pregnancy Gestation: 11 weeks 0 days; fetal heartbeat: present; CRL: 41.5 mm CRL: crown-rump length

She was admitted for further investigation. Blood tests included a full blood count, renal function tests, liver function tests, thyroid function tests, and CA125 (Table 1). The CA125 level was markedly elevated at 1389 U/mL. The MDT review concluded that the findings were consistent with sOHSS. Based on the Golan classification, the presence of ultrasound-detected ascites was consistent with moderate (Grade 3) OHSS, although the patient remained clinically well without clinically apparent ascites or systemic complications [3]. The Golan classification categorizes OHSS into mild, moderate, and severe disease according to clinical symptoms, ovarian enlargement, ascites, and systemic complications. Mild disease is characterized by abdominal discomfort and may be accompanied by gastrointestinal symptoms and ovarian enlargement. Moderate disease includes ultrasound evidence of ascites, while severe disease is associated with clinically apparent ascites and/or hydrothorax, respiratory difficulty, and, in the most advanced cases, hemocentrifugation, coagulation abnormalities, and reduced renal perfusion [3]. In our patient, marked bilateral ovarian enlargement and ultrasound-detected ascites were consistent with moderate (Grade 3) OHSS. Despite these findings, she remained clinically well without systemic complications, supporting conservative management with close surveillance. 

**Table 1:** Blood results upon admission Hb: hemoglobin; WCC: white cell count; Plts: platelets; Na: sodium; K: potassium; Cl: chlorine; HCO_3_: bicarbonate; eGFR: estimated glomerular filtration rate; ALP: alkaline phosphatase; ALT: alanine transaminase; TSH: thyroid-stimulating hormone; AFP: alpha-fetoprotein; CA-125: cancer antigen-125; CEA: carcinoembryonic antigen; LDH: lactate dehydrogenase

Parameter	Patient value		Reference range
	Day 1	Day 29	
Full Blood Count			
Hb	135	99	115 - 165g/L
WCC	10.7	6.9	4 - 1110*9/L
Plts	321	281	150 - 400 10*9/L
Hematocrit	0.391	0.282	0.37 - 0.45L/L
Renal Profile			
Na	134	132	135 - 145mmol/L
K	4.1	4.1	3.5 - 5.0U/ml
Cl	100	98	95 - 108mmol/L
HCO_3_	24	21	22 - 28mmol/L
Urea	3.6	3.0	2.5 - 7.8mmol/L
Creatinine	44	40	45 - 84umol/L
eGFR	>90	90	>90mL/min/1.73m²
Liver Function Tests			
Bili	9	6	<21umol/L
ALP	119	89	30 - 130U/L
ALT	39	9	< 40U/L
Albumin	34	38	35 - 50g/L
Thyroid Function Tests			
TSH	1.9	-	0.2 - 5mU/L
Free T4	18.6	-	10 - 24pmol/L
Tumour Markers			
AFP	5	-	<10kU/L
CA125	1389	356	0 - 35U/mL
CEA	0.56	-	<3.5ug/L
LDH	158	-	< 250 U/L

The patient was managed conservatively with strict fluid balance monitoring, prophylactic low-molecular-weight heparin (LMWH), antiemetics, and symptom control. She remained clinically stable throughout admission and was discharged with thromboprophylaxis and interval ultrasound follow-up. A repeat ultrasound four weeks later demonstrated improving ovarian volumes, measuring 622 cc on the right and 522 cc on the left (Figures 7, 8), with complete resolution of ascites and an ongoing viable pregnancy at 15+0 weeks gestation (Figure 9). Repeat blood investigations showed normal full blood count, renal function, and liver function tests. CA125 had decreased from 1389 U/mL to 356 U/mL (Table 1). The patient subsequently attended for her anomaly scan at 20+0 weeks gestation (Figures 10, 11), which demonstrated a structurally normal fetus with persistent but improving bilateral multicystic ovaries. The right ovary measured 132 × 55.1 × 51 mm with a volume of 194.6 cc, while the left ovary measured 104 × 78 × 53 mm with a volume of 226.8 cc. No ascites was present. Repeat CA125 levels continued to decline to 55 U/mL, then to 47 U/mL one week later.

**Figure 7:**
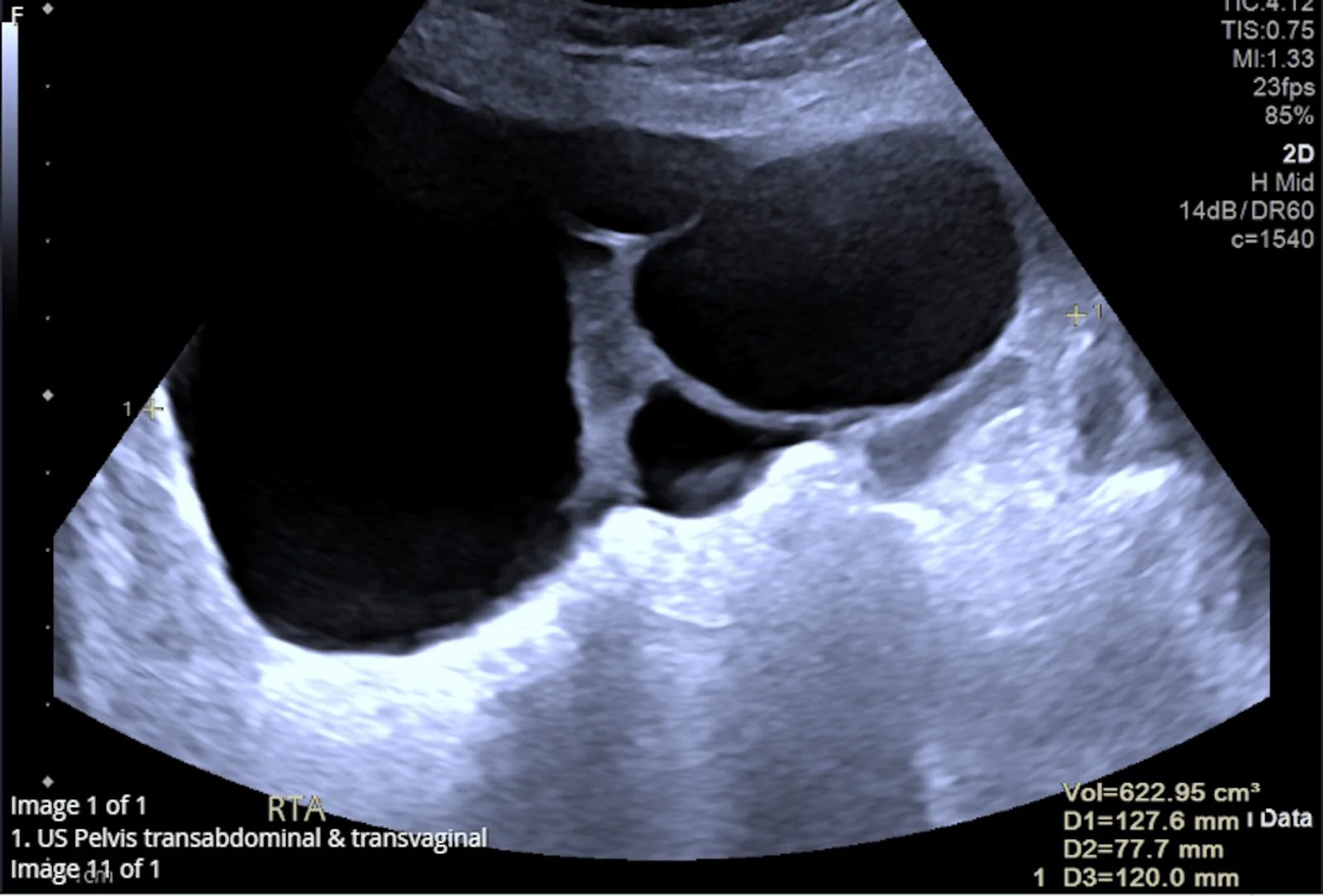
Right ovary on ultrasound scan at 15+0 weeks Right ovary with an ovarian volume of 622.95 cc, showing a reduction in ovarian volume. No ascites seen in any quadrant.

**Figure 8:**
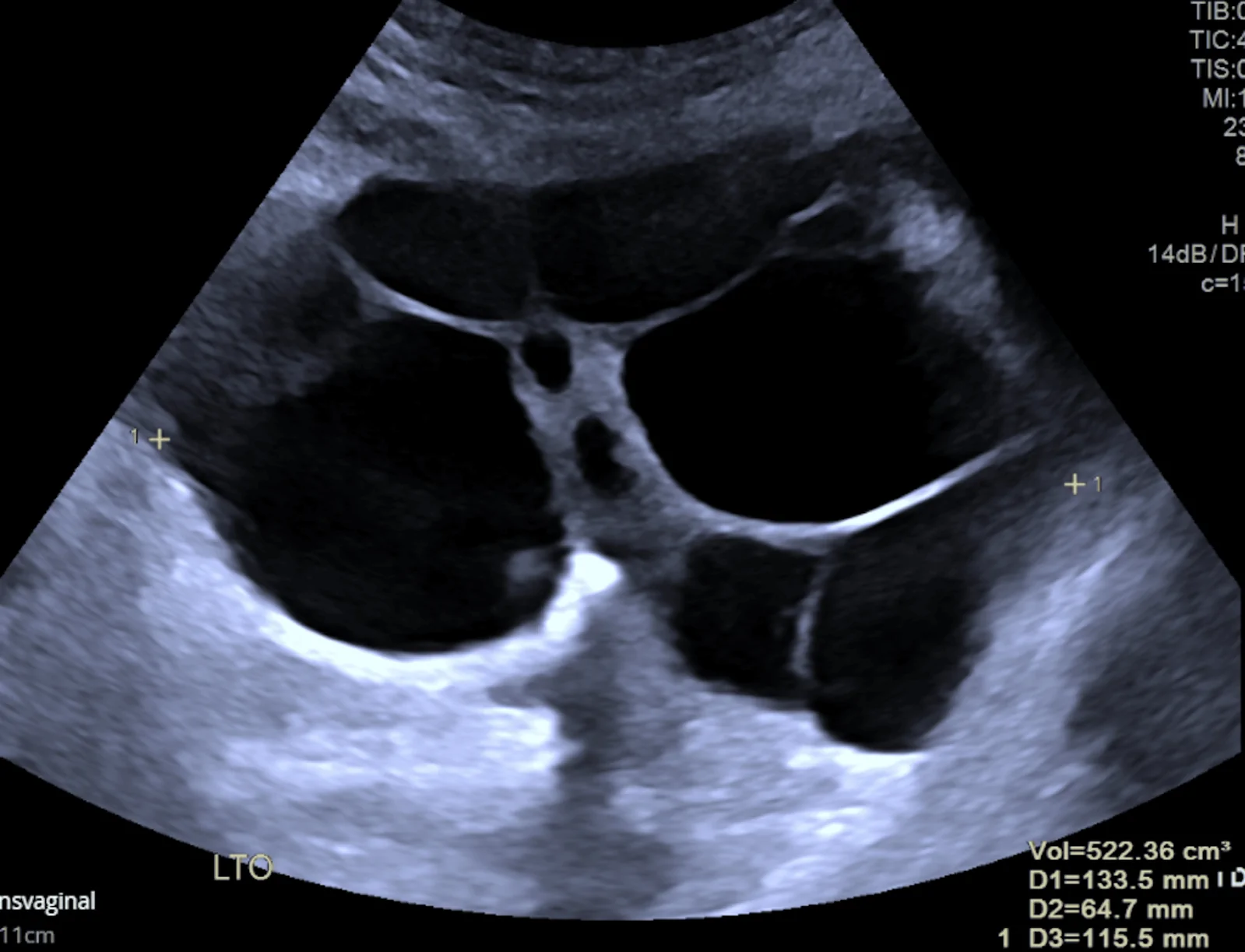
Left ovary on ultrasound scan at 15+0 weeks Left ovary with an ovarian volume of 522.36 cc, showing a reduction in ovarian volume. No ascites seen in any quadrant.

**Figure 9:**
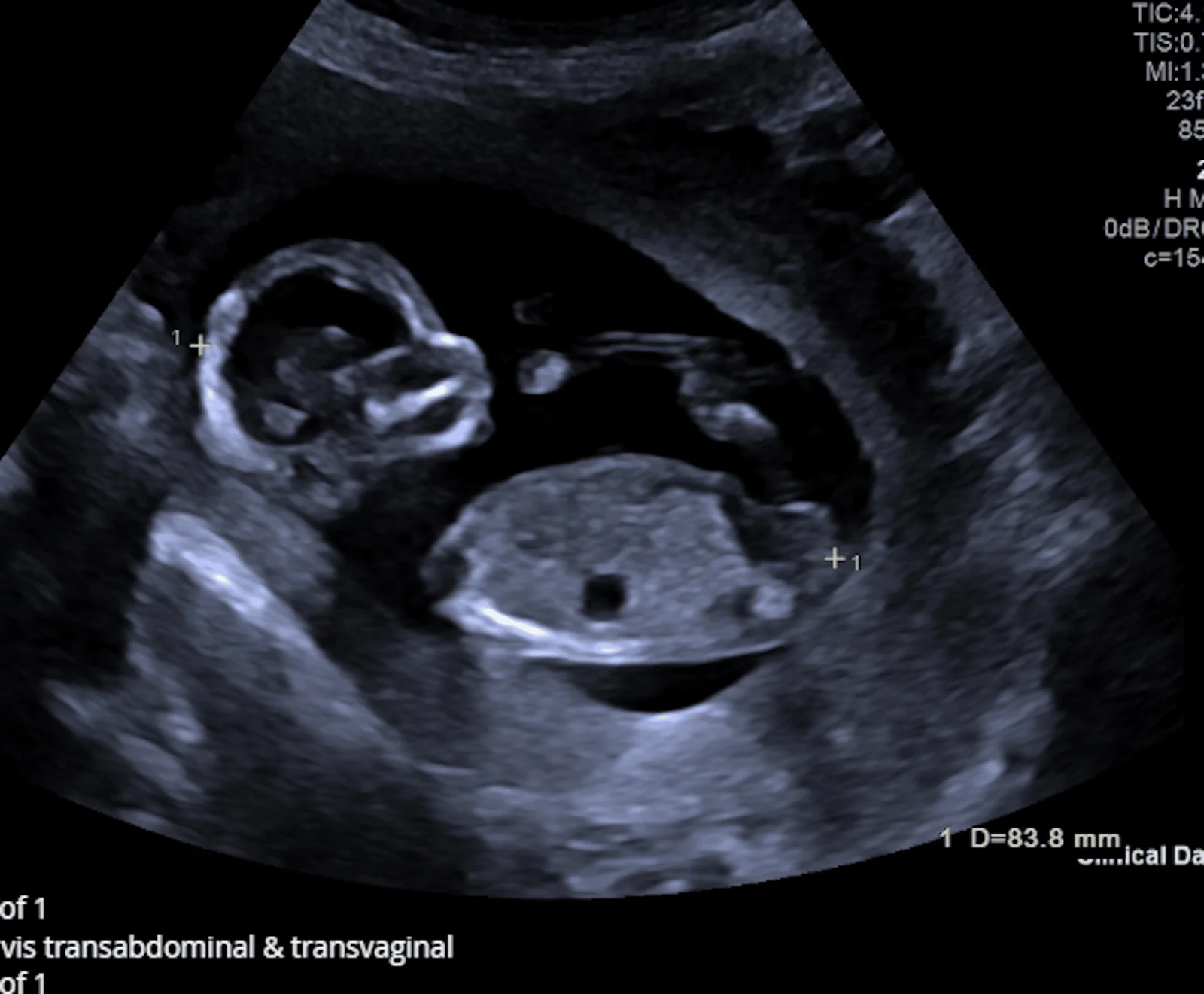
Ultrasound scan at 15+0 weeks confirming a viable intrauterine pregnancy Gestation: 15 weeks 0 days; fetal heartbeat: present; CRL: 83.8 mm CRL: crown-rump length

**Figure 10:**
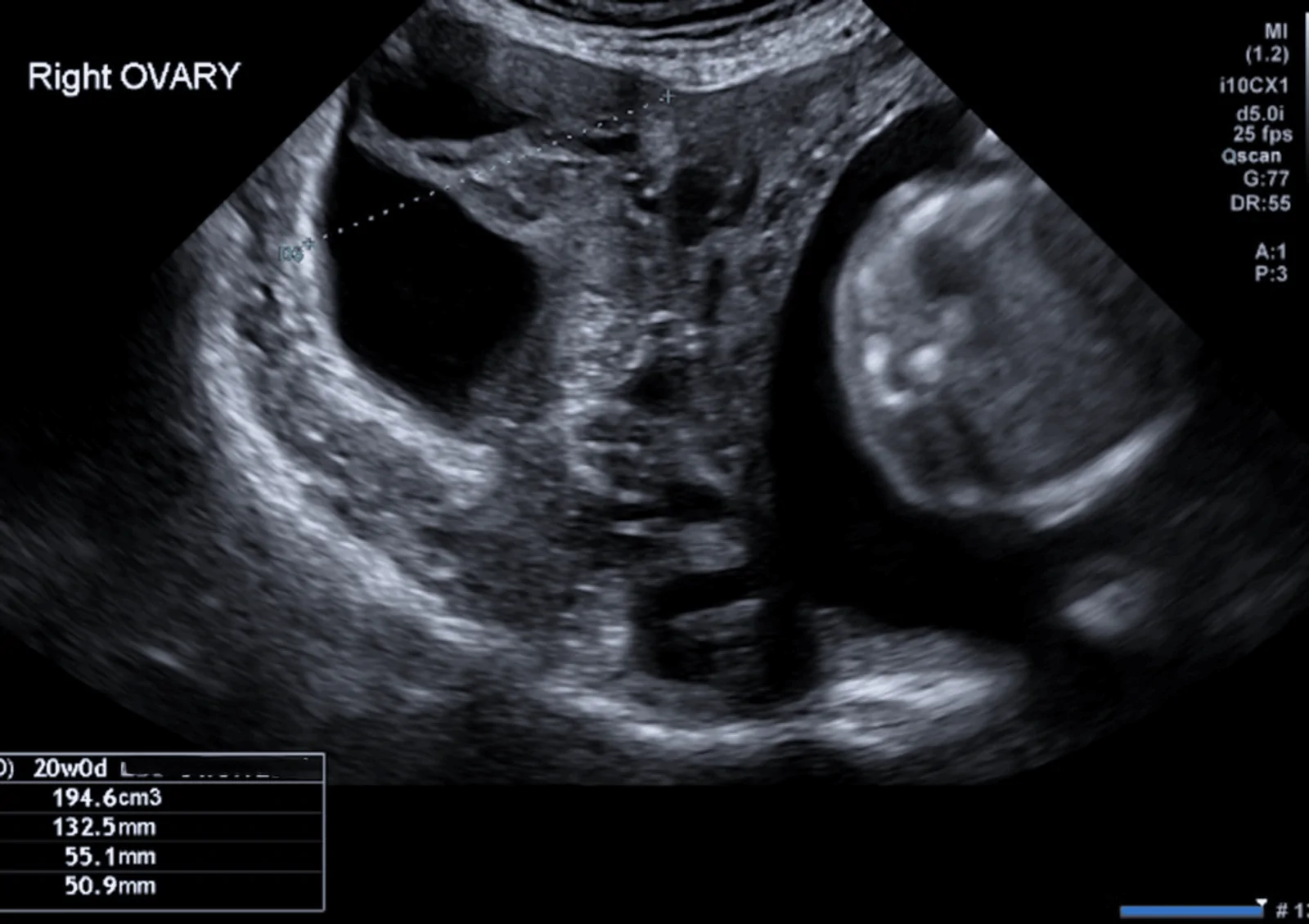
Right ovary on ultrasound at the 20-week anomaly scan Fetal heartbeat: present; normal fetal anomaly scan. Both ovaries again appear multicystic; however, the volumes have decreased since the last scan. Right ovary measures 132 x 55.1 x 51 mm with a volume of 194.6 cc (previously 622.9 cc). No ascites seen on today's scan.

**Figure 11:**
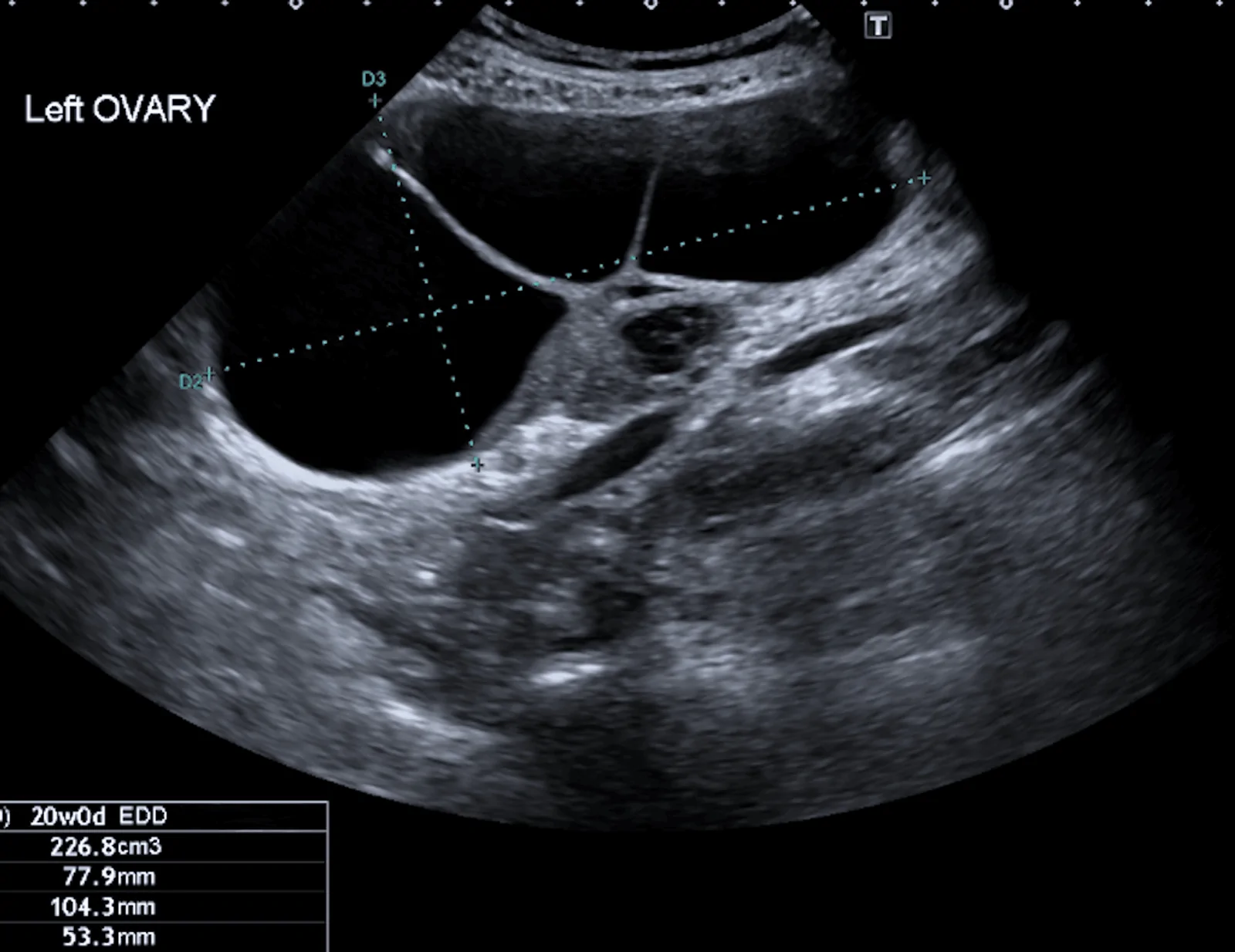
Left ovary on ultrasound at the 20-week anomaly scan Gestation: 20 weeks 0 days; normal fetal anomaly scan. Both ovaries again appear multicystic; however, the volumes have decreased since the last scan. Left ovary measures 104 x 78 x 53 mm with a volume of 226.8 cc (previously 522.3 cc). No ascites seen on today's scan.

## Discussion

sOHSS remains an exceptionally rare condition, particularly in women with spontaneously conceived singleton pregnancies and no history of assisted reproductive treatment. Unlike iatrogenic OHSS, which develops following controlled ovarian stimulation during ART, sOHSS occurs in the absence of fertility treatment and is usually encountered during the late first trimester when endogenous hCG concentrations peak. Although the true incidence remains unknown, relatively few cases have been reported, making diagnosis challenging in women without recognized risk factors [2,9,10].

The pathophysiology of OHSS is largely mediated by VEGF, which increases capillary permeability, resulting in intravascular fluid depletion, third-space fluid accumulation, ascites, and ovarian enlargement [1,11]. While this mechanism is well recognized in iatrogenic OHSS, spontaneous disease is believed to result from abnormal ovarian sensitivity to physiological concentrations of hCG. Proposed mechanisms include multiple gestation, gestational trophoblastic disease, severe hypothyroidism, and activating FSHR mutations, which permit hCG or thyroid-stimulating hormone to activate the receptor without exogenous gonadotrophin stimulation [2,4,6,12].

According to the Golan classification [3], the marked bilateral ovarian enlargement and ultrasound-detected ascites were consistent with moderate (Grade 3) OHSS. Despite these findings, the patient remained clinically well without systemic complications, supporting conservative management with close surveillance.

A notable feature of our patient was presentation at 8+5 weeks' gestation with rapidly enlarging bilateral multicystic ovaries and ascites despite a spontaneously conceived singleton pregnancy. Most published cases present between eight and 14 weeks, coinciding with rising endogenous hCG concentrations [4,9]. The absence of fertility treatment, multiple pregnancies, gestational trophoblastic disease, and hypothyroidism prompted consideration of an inherited mechanism. The strong family history of sOHSS occurring in naturally conceived pregnancies makes an FSHR-related predisposition biologically plausible. Vasseur et al. described familial sOHSS associated with a heterozygous activating FSHR mutation, while De Leener et al. demonstrated additional mutations capable of increasing receptor sensitivity to hCG and, in some cases, thyroid-stimulating hormone [5,6]. Although molecular testing was not undertaken in our patient, her presentation resembles previously published familial cases and supports consideration of postpartum genetic counseling and FSHR mutation analysis.

One of the principal diagnostic challenges was differentiating sOHSS from ovarian malignancy. The combination of rapidly enlarging bilateral multicystic ovaries, abdominal ascites, and a serum CA125 concentration of 1389 U/mL understandably raised concern for an ovarian neoplasm. The patient therefore underwent comprehensive biochemical investigation and MDT review before conservative management was recommended. The normal CEA, AFP, and LDH results were considered alongside the bilateral multicystic ovarian morphology, serial imaging, and clinical course during MDT assessment, rather than relying on CA125 alone to establish or exclude malignancy. Similar diagnostic dilemmas have been described when OHSS or pregnancy-related ovarian lesions present with ovarian enlargement, ascites, and increased CA125 [7]. This case reinforces the importance of integrating the clinical history, detailed ultrasound morphology, serial imaging, and biochemical trends before considering operative intervention.

The markedly elevated CA125 concentration represented another important finding. CA125 may increase during the first trimester of normal pregnancy, although markedly elevated values remain uncommon in women without ovarian pathology [8]. Elevated CA125 has also been documented in OHSS, particularly where ascites and peritoneal irritation are present [13,14]. In our patient, the decline in CA125 from 1389 U/mL to 47 U/mL paralleled the reduction in ovarian size and complete resolution of ascites. This trend supported a benign, self-limiting process consistent with sOHSS rather than ovarian malignancy.

Management of sOHSS is largely supportive and should be individualized according to severity. Conservative treatment includes careful fluid management, thromboprophylaxis where indicated, symptom control, and serial maternal and fetal assessment; surgery is generally reserved for complications such as ovarian torsion, rupture, or hemodynamic instability [2,15,16]. Despite substantial ovarian enlargement and ascites, our patient remained clinically stable and responded well to conservative management, with progressive clinical, biochemical, and radiological resolution. This favorable course is consistent with other reports showing that early diagnosis and multidisciplinary surveillance can avoid unnecessary surgery while preserving favorable maternal and fetal outcomes [9,10,15].

The principal limitation of this report is the absence of genetic confirmation of an FSHR mutation and the lack of serum VEGF measurement. The reported family history nevertheless makes inherited receptor hypersensitivity plausible, while the characteristic presentation, exclusion of alternative causes, and spontaneous resolution support the clinical diagnosis. Genetic testing could provide useful information regarding recurrence and future pregnancy counseling, but the absence of testing means that a specific familial mutation cannot be assumed.

Overall, this case adds to the limited literature describing sOHSS in naturally conceived singleton pregnancies. It demonstrates that marked bilateral ovarian enlargement, ascites, and significantly elevated CA125 do not necessarily indicate malignancy. A detailed family history and timely MDT review were central to the diagnosis and supported a conservative approach that avoided unnecessary surgical intervention.

## Conclusions

sOHSS in naturally conceived singleton pregnancies is rare and may mimic ovarian malignancy because of significant ovarian enlargement, ascites, and elevated CA125 levels. Early recognition, multidisciplinary involvement, and conservative management are essential in optimizing maternal and fetal outcomes while avoiding unnecessary intervention during pregnancy. The reported strong family history in this case further supports a possible inherited FSHR-related predisposition. Genetic counseling and consideration of FSHR mutation testing may therefore be beneficial in assessing familial risk and informing future pregnancy counseling.
